# *In Situ* Patrolling of Regulatory T Cells Is Essential for Protecting Autoimmune Exocrinopathy

**DOI:** 10.1371/journal.pone.0008588

**Published:** 2010-01-05

**Authors:** Naozumi Ishimaru, Takeshi Nitta, Rieko Arakaki, Akiko Yamada, Martin Lipp, Yousuke Takahama, Yoshio Hayashi

**Affiliations:** 1 Department of Oral Molecular Pathology, Institute of Health Biosciences, The University of Tokushima Graduate School, Kuramotocho, Tokushima, Japan; 2 Department of Experimental Immunology, Institute for Genome Research, The University of Tokushima Graduate School, Kuramotocho, Tokushima, Japan; 3 Department of Molecular Tumor Genetics and Immunogenetics, Max-Delbruck Center for Molecular Medicine, Berlin, Germany; New York University, United States of America

## Abstract

**Background:**

Migration of T cells, including regulatory T (Treg) cells, into the secondary lymph organs is critically controlled by chemokines and adhesion molecules. However, the mechanisms by which Treg cells regulate organ-specific autoimmunity via these molecules remain unclear. Although we previously reported autoimmune exocrinopathy resembling Sjögren's syndrome (SS) in the lacrimal and salivary glands from C-C chemokine receptor 7 (CCR7)-deficient mice, it is still unclear whether CCR7 signaling might specifically affect the dynamics and functions of Treg cells *in vivo*. We therefore investigated the cellular mechanism for suppressive function of Treg cells via CCR7 in autoimmunity using mouse models and human samples.

**Methods and Findings:**

Patrolling Treg cells were detected in the exocrine organs such as lacrimal and salivary glands from normal mice that tend to be targets for autoimmunity while the Treg cells were almost undetectable in the exocrine glands of *CCR7* ^−/−^ mice. In addition, we found the significantly increased retention of CD4^+^CD25^+^Foxp3^+^ Treg cells in the lymph nodes of *CCR7* ^−/−^ mice with aging. Although Treg cell egress requires sphingosine 1-phosphate (S1P), chemotactic function to S1P of *CCR7*−/− Treg cells was impaired compared with that of WT Treg cells. Moreover, the *in vivo* suppression activity was remarkably diminished in *CCR7* ^−/−^ Treg cells in the model where Treg cells were co-transferred with *CCR7* ^−/−^ CD25^-^CD4^+^ T cells into *Rag2* ^−/−^ mice. Finally, confocal analysis showed that CCR7^+^Treg cells were detectable in normal salivary glands while the number of CCR7^+^Treg cells was extremely decreased in the tissues from patients with Sjögren's syndrome.

**Conclusions:**

These results indicate that CCR7 essentially governs the patrolling functions of Treg cells by controlling the traffic to the exocrine organs for protecting autoimmunity. Characterization of this cellular mechanism could have clinical implications by supporting development of new diagnosis or treatments for the organ-specific autoimmune diseases such as Sjögren's syndrome and clarifying how the local immune system regulates autoimmunity.

## Introduction

Emerging evidence demonstrates that CD4^+^CD25^+^Foxp3^+^ regulatory T cells (Treg cells) play a central role in the protection of autoimmunity [1]–[3]. Treg cells actively suppress pathological and physiological immune responses, contributing to the maintenance of immunological self-tolerance and immune homeostasis. However, it has not been clarified whether the ability of Treg cells to migrate among tissues is important for them in exerting their suppressor function. On the other hand, recent reports showed that Treg cells are also present within non-lymphoid sites in the periphery, including autoimmune lesions, infectious sites, and tumors [4]. The depletion of Treg cells from normal mice leads to spontaneous development of various autoimmune diseases, such as autoimmune thyroiditis, type 1 diabetes, gastritis, and inflammatory bowel disease [5]–[8]. Although the injection of Treg cells into animal models for autoimmunity can prevent or reduce the onset of diseases [9]–[11], it is still unclear which sites of Treg cells can display their function *in vivo*. In a model of type I diabetes, NOD mice, the disease resistance has been correlated with the expansion of Treg cells within inflamed pancreatic lymph nodes [12]. Moreover, it was demonstrated that the number of Treg cells increased in the lamina propria of inflamed tissues from patients with inflammatory bowel disease (IBD) while the number of Treg cells is significantly reduced in the peripheral blood mononuclear cells (PBMCs) from the patients [8], [13], [14]. Although various molecular and cellular events have been described to explain the mechanisms of Treg-mediated suppression [15], [16], none of the proposed mechanisms can explain all aspects of *in vivo* suppression.

Regarding to the nature of the molecules that may be involved in the trafficking of Treg cells to lymphoid and non-lymphoid sites, the participation of chemokine receptors has been demonstrated by several studies [17]–[19]. Recent studies suggest that C-C-chemokine receptor 7 (CCR7) and its ligands are involved in the migration of naive T cells into the lymph nodes (LNs) and play an important role in the initiation of adaptive immune responses [20]–[24]. It has been shown that Treg cells can change their chemotactic behavior by switching the expression of CXCR5 and CCR7 in response to the ligands, CXCL13 and CCL19 respectively [25]. Previously we reported that autoimmune exocrinopathy resembling Sjögren's syndrome (SS) developed in *CCR7* ^−/−^ mice [26]. In addition, it was indicated that enhanced immunity in *CCR7* ^−/−^ mice is caused by the defective lymph node positioning of Treg cells and the consequent impediment of the suppressor function [23]. However, it is still unclear whether CCR7 signaling might specifically affect the dynamics and functions of Treg cells *in vivo*. We thus examined the role of CCR7 signaling in the *in vivo* function of Treg cells that regulate the development of autoimmunity.

## Results

### Patrolling Treg Cells in the Exocrine Glands

**Figure 1:**
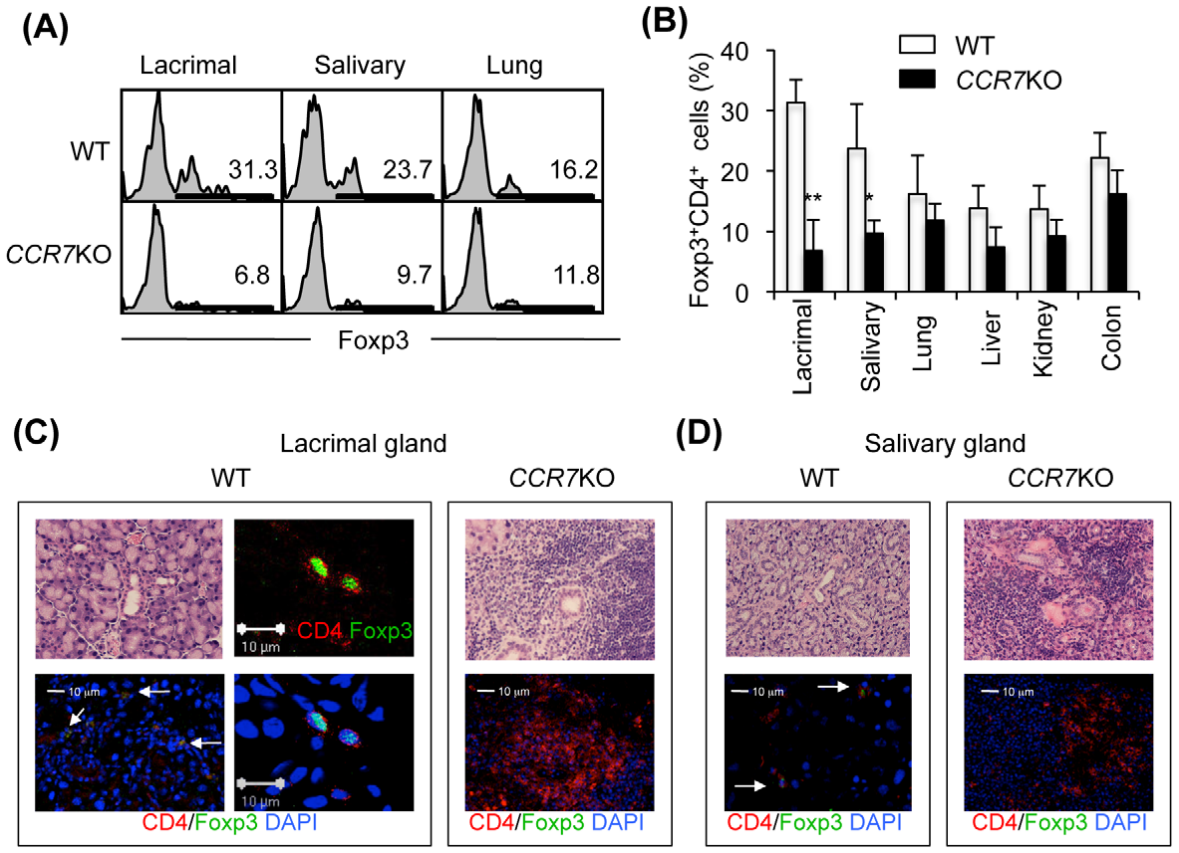
Tissue Distribution of Treg Cells. (A) Flow cytometric analysis of Foxp3^+^CD4^+^ T cells in tissue-infiltrating lymphocytes was performed using lacrimal, salivary glands, and lung of WT and *CCR7* ^−/−^ mice. (B) Foxp3^+^CD4^+^ Treg cells (%) were shown using lacrimal, salivary glands, lung, liver, kidney, and colon from WT and *CCR7* ^−/−^ mice. Data are means±s.e.m. of 3 to 4 mice per each group. *p<0.05, **p<0.005, versus WT. (C and D) Foxp3^+^CD4^+^Treg cells were detected on the frozen sections of lacrimal and salivary glands from WT and *CCR7* ^−/−^ mice. Upper photos show histology stained with H&E. Foxp3 and CD4 are shown as green and red. Nuclei were stained with DAPI (blue). Results are representative of 3 to 4 mice per each group.

We have reported that the onset of autoimmune lesions in lacrimal and salivary glands of *CCR7* ^−/−^ mice was at around 10 weeks of age [26]. It is still unclear whether Treg cells infiltrate into tissues which include the lacrimal or salivary glands as the target organs in *CCR7* ^−/−^ mice. Therefore, we tested for tissue-infiltrating Treg cells in various tissues including lacrimal, salivary glands, lung, liver, kidney and colon of WT and *CCR7* ^−/−^ mice using flow cytometric analysis with Foxp3 expression. Interestingly, the Treg cells within the lacrimal and salivary glands of *CCR7* ^−/−^ mice were significantly decreased in comparison with those of WT mice (Fig. 1A and B). By contrast, there was no difference in the Treg cells within the other organs between WT and *CCR7* ^−/−^ mice (Fig. 1B). When immunofluorescence staining with anti-CD4 and Foxp3 mAbs was performed using frozen sections of the salivary and lacrimal glands from WT and *CCR7* ^−/−^ mice, Foxp3^+^CD4^+^ Treg cells were found in the lacrimal and salivary gland tissues from normal WT mice although the cell number was lower (Fig. 1C and D). To the contrary, Foxp3^+^ Treg cells were almost undetectable in the lacrimal and salivary gland tissues of *CCR7* ^−/−^ mice (Fig. 1C and D). Thus, the presence of “patrolling” Treg cells within tissues such as the lacrimal and salivary glands is associated with the maintenance of peripheral tolerance to protect autoimmunity.

### Treg Cells in the Lymphoid Tissues of CCR7^−/−^ Mice

**Figure 2:**
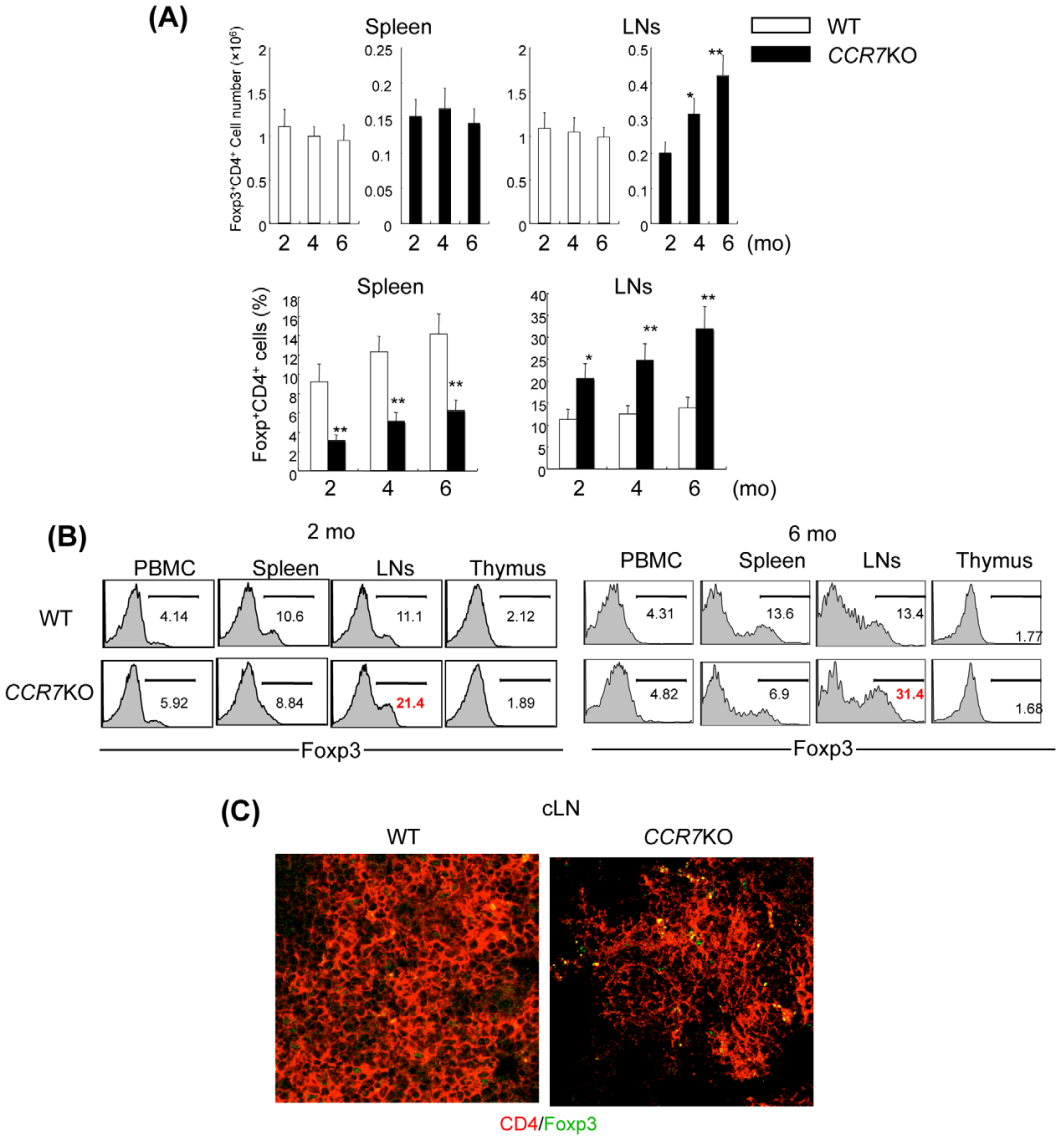
Change of Treg Cells with Aging in Spleen and LNs of *CCR7* ^−/−^ Mice. (A) The number of Foxp3^+^CD4^+^ T cells in spleen and LNs from WT and *CCR7* ^−/−^ mice from 2 to 6 months of age was analyzed by flow cytometer. Data are means±s.e.m. of 7 to 10 mice in each group. *p<0.05, versus the cell number at 2 mo (upper panel), *p<0.05, **p<0.005, versus WT. (B) Foxp3 expressions of CD4^+^ T cells in PBMC, spleen, LNs, and thymus from WT and *CCR7* ^−/−^ mice at 2 and 6 months of age were analyzed by flow cytometer. (C) CD4^+^Foxp3^+^ cells in cervical lymph node were analyzed by confocal microscopy. Figures and photos are representative of 3 to 7 mice in each group.

In order to examine the basis for the decreased number of Treg cells in the target organs of autoimmunity in *CCR7* ^−/−^ mice, Treg cells in lymphoid organs between WT and *CCR7* ^−/−^ mice were compared using flow cytomtric analysis. Although the absolute number of T cells in the spleen and lymph nodes (LNs) from *CCR7* ^−/−^ mice was found to be significantly decreased compared with that from WT mice as previously described [23], a significant increase in *CCR7* ^−/−^ Treg cell retention with aging was observed in LNs, not in the spleen (Fig. 2A). The population of Foxp3^+^ cells (%) among CD4^+^ cells in LNs of *CCR7* ^−/−^ mice was found to be significantly increased compared with that of WT mice while the population was significantly decreased in the spleen (Fig. 2A and B). There was no difference of Foxp3^+^ cells (%) in PBMCs and thymocytes between *CCR7* ^−/−^ and WT mice (Fig. 2B). In addition, an increase in Foxp3^+^CD4^+^Treg cells in the cervical LNs (cLN) of *CCR7* ^−/−^ mice was observed, in comparison with that in WT cLNs at 6-months-old (Fig. 2C). On the other hand, CD25^−^CD4^+^ T cells from *CCR7* ^−/−^ mice were not accumulated both in spleen and cLNs (Fig. S1). These findings suggest that the impaired traffic of *CCR7* ^−/−^ Treg cells from the regional lymph nodes to target organs of autoimmunity influences the development of organ-specific autoimmunity.

### Migration of CCR7^−/−^ Treg Cells

**Figure 3:**
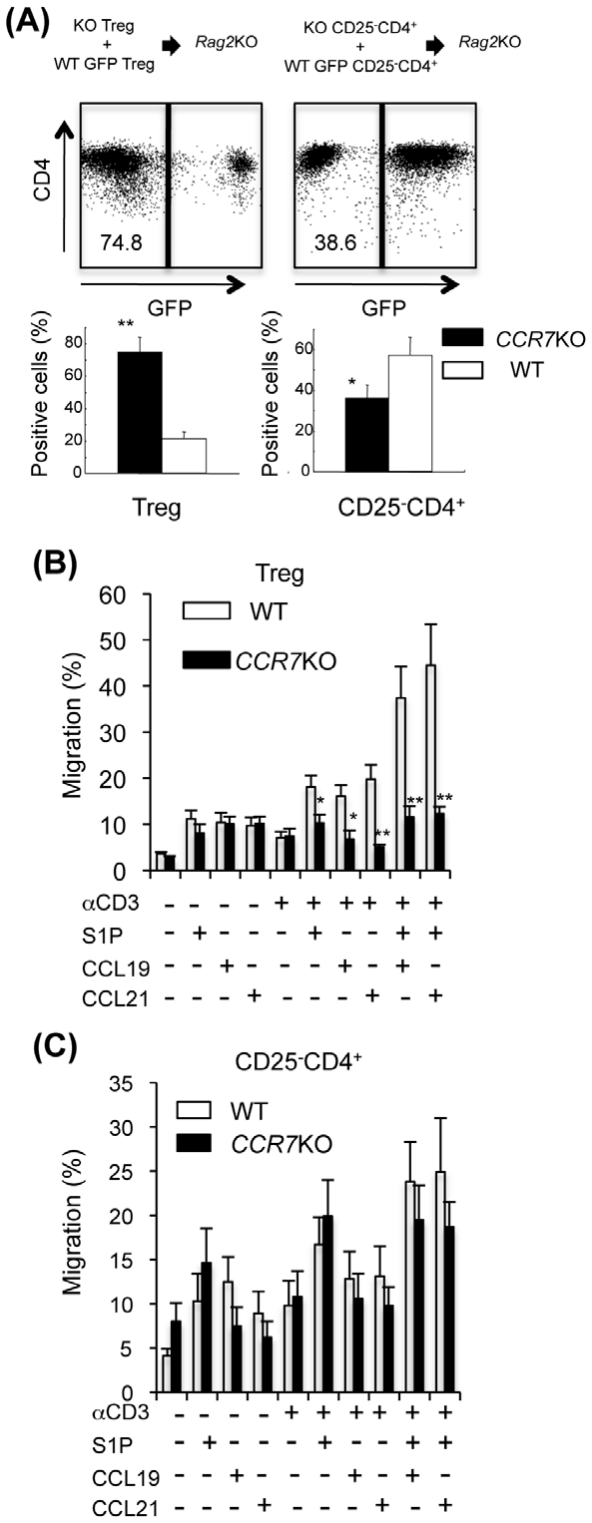
Homing and Egress of *CCR7* ^−/−^ Treg Cells. (A) *CCR7* ^−/−^ Treg cells (1×10^6^) and WT GFP-Tg Treg cells (1×10^6^), or *CCR7* ^−/−^ CD25^-^CD4^+^ and WT CD25^-^CD4^+^ cells were co-transferred into *Rag2* ^−/−^ mice. At 2 weeks later CD4^+^ GFP^−^ cells (from *CCR7* ^−/−^ mice) or CD4^+^ GFP^+^ cells (from WT mice) in LNs of the recipient *Rag2* ^−/−^ mice were detected by flow cytometry. Results are representative of five to seven mice in each group. *p<0.05, **p<0.005, versus WT. (B) Migration assay of Treg cells from LNs of WT and *CCR7* ^−/−^ mice was performed with anti-CD3 mAb, S1P, CCL19 and CCL21. Data are means±s.e.m. of triplicate and are representative of three independent experiments. *p<0.05, **p<0.005, versus WT.

It is unclear whether the *in vivo* homing of *CCR7* ^−/−^ Treg cells to LNs is impaired. To examine the *in vivo* movement of Treg cells, Treg cells from *CCR7* ^−/−^ mice were co-transferred with WT green fluorescence protein (GFP)-transgenic (Tg) Treg cells into *Rag2* ^−/−^ mice. We found the significantly increased accumulation of *CCR7* ^−/−^ Treg cells in the lymph nodes compared with WT Treg cells (Fig. 3A), indicating that the absence of CCR7 on Treg cells may influence the egress from LNs rather than the homing to LNs.

On the other hand, T cell egress from the thymus and peripheral lymphoid organs depends on sphingosine 1-phosphate (S1P) receptor-1 (S1P_1_) signaling, and is thought to occur in response to circulatory S1P [27]–[30]. Although recent work has shown that S1P is required for induction of egress [31], [32], the mechanism by which S1P_1_ acts to regulate the traffic of Treg cells has been unclear. To examine whether the egress of *CCR7* ^−/−^ Treg cells from LNs is impaired, *in vitro* migration assay to S1P or CCR7's ligands using a boyden chamber with polyethylene terephthalate membrane was performed. We found that CD3-engaged LN Treg cells in *CCR7* ^−/−^ mice could not migrate to S1P, while Treg cells from WT mice efficiently migrated to S1P upon CD3 engagement (Fig. 3B). When the ligand of CCR7, CCL19 or CCL21, was added to the culture of CD3-engaged Treg cells with or without S1P, the migratory responses of WT Treg cells were highly enhanced compared with the responses of *CCR7* ^−/−^ Treg cells (Fig. 3B). In contrast, the migration activity to S1P upon CD3 engagement of *CCR7* ^−/−^ non-Treg cells was equivalent to that of WT non-Treg cells (Fig. 3C). These results indicate that CCR7 crucially regulates the egress of Treg cells from the LNs.

### Impaired In Vivo Suppressive Function of CCR7^−/−^ Treg Cell

**Figure 4:**
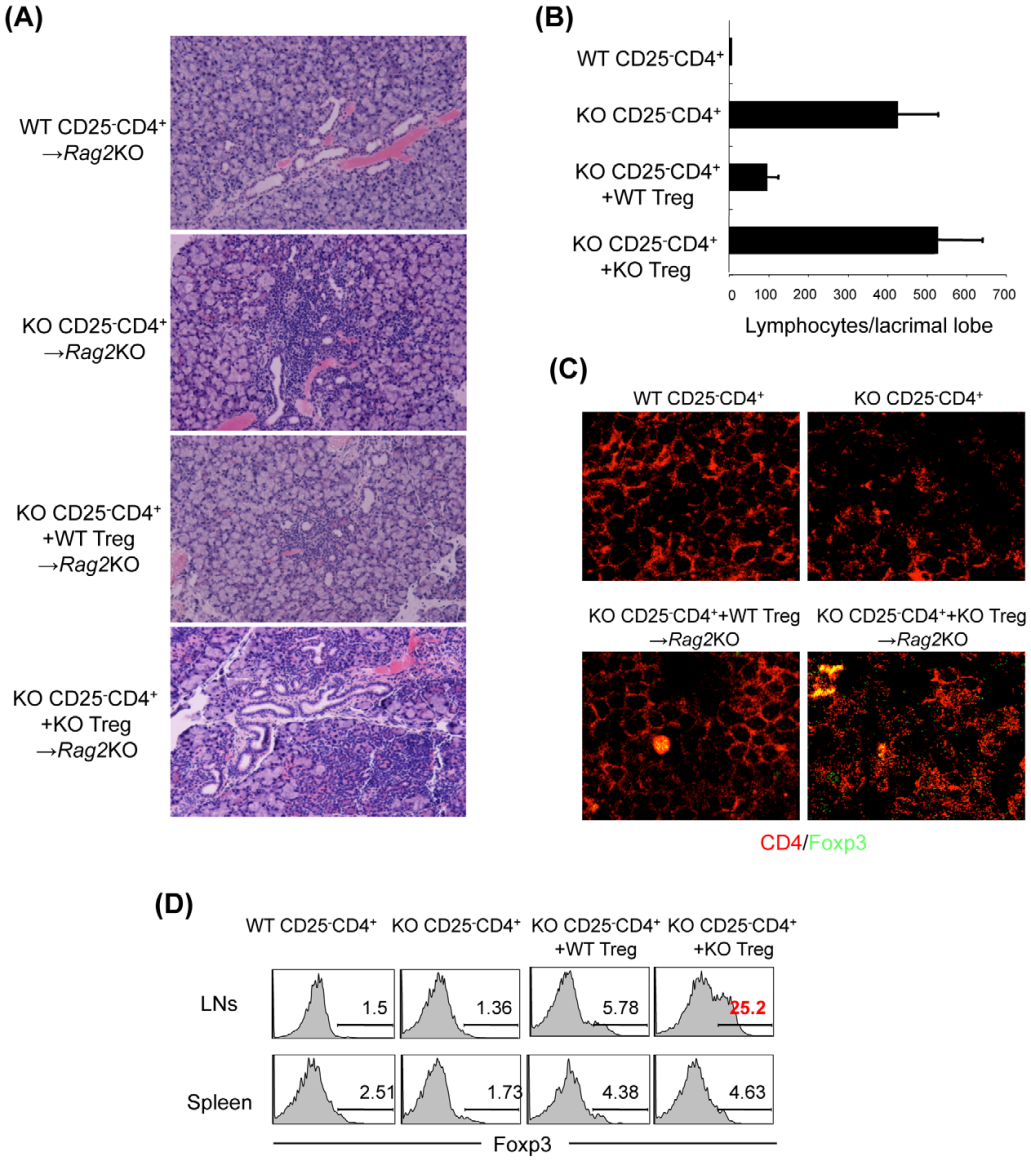
Disorder of *In Vivo* Regulatory Function of Treg Cells in *CCR7* ^−/−^ Mice. (A) WT or *CCR7* ^−/−^ CD25^−^CD4^+^ T cells (5×10^6^) were transferred into *Rag2* ^−/−^ mice. WT or *CCR7* ^−/−^ Treg cells (2.5×10^6^) were co-transferred with *CCR7* ^−/−^ CD25^−^CD4^+^ T cells (5×10^6^) into *Rag2* ^−/−^ mice. At 6 week after the transfer all organs were histopathologically analyzed. Pathology of lacrimal glands from the *Rag2* ^−/−^ recipient mice was shown using the sections stained with hematoxylin and eosin. (B) Histological evaluation of inflammatory lesions in the recipient *Rag2* ^−/−^ mice. Data are means±s.e.m. of 5 to 7 mice in each group. (C) Foxp3^+^CD4^+^ Treg cells in lymph nodes were detected by confocal analysis. (D) Foxp3 expression of CD4^+^ T cells in LNs and spleen was analyzed by flow cytometer. *p<0.05, **p<0.005, versus WT.

In order to evaluate the function of *CCR7* ^−/−^ Treg cells *in vivo*, we transferred Treg cells from *CCR7* ^−/−^ and WT mice into mice that exhibited inflammatory lesions in lacrimal and salivary glands resembling SS by the transfer of *CCR7* ^−/−^ CD25^−^CD4^+^ LN T cells into *Rag2* ^−/−^ mice (Fig. 4A and B). Upon the co-transfer of WT Treg cells, the lesions of lacrimal glands were clearly prevented (Fig. 4A and B). However, the co-transfer of *CCR7* ^−/−^ Treg cells did not prevent the autoimmune lesions (Fig. 4A and B). We also found that the number of Foxp3^+^CD4^+^Treg cells was increased in cervical lymph nodes (cLNs) but not the spleen of *Rag2* ^−/−^ mice transferred with *CCR7* ^−/−^ Treg cells compared with those transferred with WT Treg cells (Fig. 4C and D). These results suggested that CCR7 expression on Treg cells influence the *in vivo* mechanism for the development of autoimmune exocrinopathy.

### CCR7^+^ Treg Cells in the Human Target Organ

**Figure 5:**
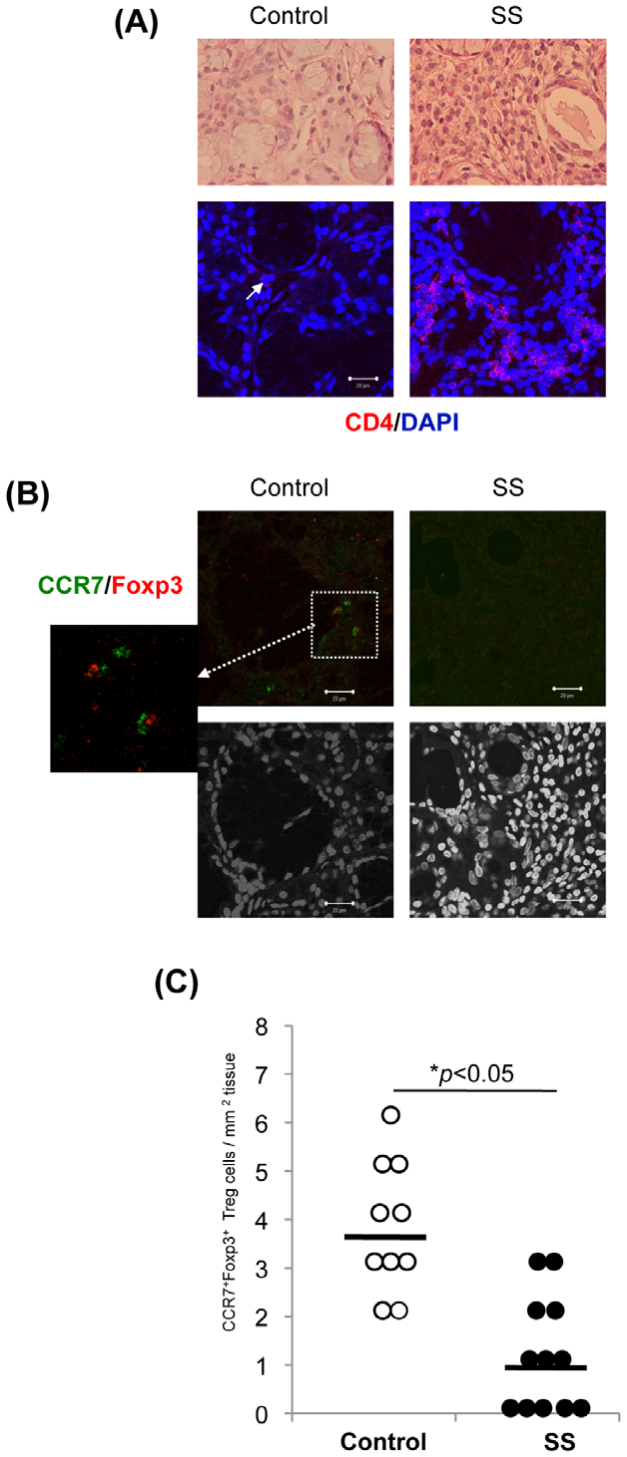
CCR7^+^Treg Cells in the Human Salivary Gland Tissues. (A) Histology of salivary gland tissues in SS patients. The sections of lip biopsy from controls and SS patients were stained with H&E. Photos are representative of 5 to 7 samples. (B) CD4^+^ T cells in the salivary gland tissues were detected by immunofluorescence staining. Nuclei were attained with DAPI. Photos are representative of 7 to 10 samples. (C) CCR7^+^Foxp3^+^ cells were detected in the frozen sections of salivary glands from controls and SS patients. Photos are representative of 10 to 12 samples. (D) The cell number of CCR7^+^Foxp3^+^ cells in the salivary gland tissues was evaluated. The results are shown as the number per 1 mm^2^ of the tissues from 10 controls and 12 SS patients. *p<0.05, control versus SS.

To examine whether the patrolling Treg cells are observed in human salivary gland tissues, CCR7^+^Foxp3^+^ Treg cells of the salivary glands from controls and SS patients were analyzed using sections of lip biopsy samples. The histopathological finding of salivary gland tissues from patients with SS showed the destruction or atrophy of acinar cells, and lymphocyte infiltration around ductal cells while there was no inflammation in the tissues from controls (Fig. 5A). However, a small number of lymphocytes were detectable in the stromal tissue of salivary glands from controls (Fig. 5A). The major population of infiltrating immune cells in the salivary gland tissues from SS patients was CD4^+^ T cell (Fig. 5B). Although the cell number of lymphocyte in control tissues was considerably lower, a few CD4^+^ T cells were detectable (Fig. 5B). In addition, CCR7^+^Foxp3^+^ Treg cells were detectable in the connective tissue and around the acinar or ductal cells of control salivary glands while the cells were hardly detected among the inflammatory lesions of SS patients (Fig. 5C). Approximately 40∼50% cells among infiltrating immune cells in the salivary glands from SS patients was CD4^+^ as shown in Figure 5A. The reduction of Treg cell number in the salivary glands from SS patients seemed to be not related to an overall reduction of CD4^+^ T cells. When the cell number of CCR7^+^Foxp3^+^ Treg cells in the control samples was compared with that from SS patients, the number of controls (3.70±1.34/mm^2^) was significantly increased than that of SS patients (1.18±1.16/mm^2^) (Fig. 5D). The reduction of Treg cell number in the salivary glands from SS patients seemed to be not related to an overall reduction of CD4+ T cells. A small number of CCR7^+^Foxp3^−^ cells in the salivary gland tissues from SS patients were also found (Fig. S2). However, the CCR7^+^Foxp3^−^ cells were hardly detectable in the control tissues (data not shown). As it is known that CCR7 is expressed on T cells and mature dendritic cells [33], the cells expressing CCR7 in the inflamed salivary gland tissues from SS patients may be dendritic cells or T cells other than Treg cells. This finding showed that CCR7^+^ Treg cells are patrolling within the target organ such as salivary gland to protect human autoimmunity as well as a murine model.

## Discussion

Our previous report demonstrated that CCR7 plays an essential role in the migration of thymocytes from the cortex to the medulla in the thymus for maintaining central immune tolerance [26]. It was suggested that mature thymocytes exported from the cortex in the absence of CCR7 were potent in causing autoimmunity. We found that the autoreactive T cells inducing autoimmune lesions in *CCR7* ^−/−^ mice are generated in the thymus where positively selected thymocytes including any autoreactive T cells are exported from the cortex. However, it is still unclear how the autoimmunity is caused by the disturbance in the peripheral immune system.

It is well known that lymphocyte egress from lymphoid organs is essential for immunsurveillance [29], [31], [34]. In contrast to the cascade of molecular interactions required for cell entry from blood into tissues [35]–[37], the multistep requirements for lymphocyte egress, in particular for Treg cells, have not been defined. It has been demonstrated that Foxp3 plays an essential role in suppressing AP-1 DNA-binding activity and consequently inhibits AP-1 transcription activity, because expression of Foxp3 significantly blocked AP-1 transcription activity and promoter DNA-binding [38]. In addition, it has been suggested that the migratory capacity of Treg cells is controlled by distinct signals from chemokines/chemokine receptors. Recent studies have shown that CCR7 and CCR7 ligands are not only involved in cell entry to lymphoid tissues but also promote T cell motility [38]–[40]. Additionally, our finding as to the increased accumulation of *CCR7* ^−/−^ Treg cells in the lymph nodes was different from the report that transferred *CCR7* ^−/−^ Treg cells fail to migrate into the lymph nodes and suppress antigen-induced T cell response [23]. In our experiment, the *CCR7* ^−/−^ Treg cells were transferred into *Rag2* ^−/−^ mice which are lymphopenic while *CCR7* ^−/−^ Treg cells bearing antigen-specific T cell receptor cells were transferred into normal mice in the previous report [23]. It was indicated that the phenotype and function of Treg cells were largely changed in the lymphopenic condition compared with normal one [41], [42]. It was possible that the homing function of *CCR7* ^−/−^ Treg cells might be altered in the lymphopenic condition.

As for the other T cells, *CCR7* ^−/−^ CD25^−^CD4^+^ T cells were analyzed together with the Treg cells in this study. We observed no retention of *CCR7* ^−/−^ CD25^-^CD4^+^ T cells in LNs. Thus, there are some differences in the response via CCR7 between Treg and CD25^−^CD4^+^ cells. On the other hand, the abnormal findings of *CCR7* ^−/−^ Treg cells were observed in LNs, not in the spleen. Levels of S1P and chemokines are different in the lymph nodes, blood, and various tissues [32]. It is possible that the functions of Treg cells may be dependent on the response to each level of S1P or chemokines such as CCL19 and CCL21 through S1P_1_ or CCR7. Moreover, a recent report demonstrated that CCR7-mediated donor-derived antigen-presenting cell (APC) trafficking to the draining LN is not only important in initiation of host T cell priming, but also crucial for Treg-mediated tolerance [43]. Although our study has not focused on the role of CCR7 in APCs, the expressions of CCR7 mRNA of APCs such as CD11c^+^ dendritic cells, CD11b^+^ macrophages, or B220^+^ B cells were detectable (data not shown). It is possible that CCR7 plays any important roles in APC functions in immune responses.

When we analyzed cytokine secretions of *CCR7* ^−/−^ and WT Treg cells including IL-4, IL-10, and TGF-β using the supernatants from Treg cells stimulated by plate-coated anti-CD3 and CD28 mAbs. There were no differences in the concentration levels of the cytokines between *CCR7* ^−/−^ and WT Treg cells. Moreover, we observed the in vitro, not in vivo, suppression function of *CCR7* ^−/−^ Treg cells similar to WT Treg cells as previously described [23].

Our hypothesis is that the egress of Treg from LNs into the target organs is essentially required for a suppressive effect on autoimmunity. When the Treg cells from the immune cells that had infiltrated the lacrimal and salivary glands of WT mice were evaluated by flow cytometric analysis and immunostaining, significantly increased Foxp3^+^ Treg cells were detectable compared with those of *CCR7* ^−/−^ mice. The exocrine organs such as the lacrimal and salivary glands tend to be target organs for autoimmunity in *CCR7* ^−/−^ mice. It is possible that functional Treg cells might be patrolling in the exocrine glands such as the lacrimal and salivary glands to protect autoimmunity *in vivo*. Recently, we reported that a significantly decreased number of Treg cells was found in the lamina propria (LP) of intestinal mucosa tissues of a rat model for inflammatory bowel disease compared with the Treg cell number in normal LP [44]. Thus, trafficking of Treg cells into inflammatory lesions could be crucial for *in vivo* suppression of autoimmunity. On the other hand, it was reported that antigen-specific Treg cells expanded in the peripheral lymphoid compartment and readily accumulated in the central nerve system, but not prevent the onset of the disease using experimental autoimmune encephalomyelitis (EAE), an animal model for multiple sclerosis [45]. Furthermore, it was described that high proportions of Treg cells are also found in the islets of NOD mice protected following various types of interventions [46]. Therefore, a lack of controlling autoimmunity may not always correlate with the number of Treg cells in a target tissue.

The findings of target organ from human controls and SS patients would potently support our hypothesis. The trafficking receptors on human Treg cells including CCR7 are controlled at each differentiation stage in secondary lymphoid tissues [47]. It was described that CD25^+^CCR7^+^CD62L^+^CTLA-4^+^Foxp3^+^ cell is one of peripheral circulating compartments of natural naïve CD4^+^ Treg cells [48]. In addition, our result as for human Treg cells was consistent with the previous report that CD25^high^CD4^+^T cells are markedly diminished in the PBMCs and salivary gland tissues from SS patients [49]. Although it is still unclear whether the CCR7^+^Foxp3^+^ Treg cells in the normal salivary glands are natural naïve Treg cells, the decreased cell number in SS patients suggests that the expression of CCR7 on Treg cells may play a key role in the protection of autoimmunity.

Our data provided the first evidence of an indispensable role of CCR7 on Treg cell egress from the lymph nodes. Together, CCR7-expressing Treg cells within the target tissue may control the organ-specific self-tolerance to prevent autoimmunity in the periphery. Moreover, analyzing the unique character of Treg cells would help the establishment of the new diagnosis or therapeutic strategy for human autoimmunity.

## Materials and Methods

### Ethics

This study was conducted according to the principles expressed in the Declaration of Helsinki. The study was approved by the Institutional Review Board of the University of Tokushima and Tokushima University hospital (toku09021). All patients provided written informed consent for the collection of samples and subsequent analysis.

### Mice

*CCR7* ^−/−^, *CCR7* ^+/−^, *CCR7* ^+/+^, *Rag2* ^−/−^, GFP-transgenic mice, and C57BL/6 mice were reared in our specific pathogen-free mouse colony, and given food and water ad libitum.

### Histological Analysis

All organs were removed from the mice, fixed with 4% phosphate-buffered formaldehyde (pH 7.2) and prepared for histological examination. Formalin-fixed tissue sections were subjected to hematoxylin and eosin (H&E) staining.

### Cell Preparation

Treg cells and CD25^−^CD4^+^ cells were enriched from lymph nodes or spleen. In brief, CD4^+^ cells were prepared using anti-B220, CD8, MHC class II, and NK1.1 mAbs (eBioscience, San Diego, CA) and Dynal magnetic beads (Invitrogen, Carlsbad, CA). CD25^+^CD4^+^ or CD25^−^CD4^+^ cells were enriched using biotin-conjugated anti-CD25 mAb, magnetic beads, and Dynal CELLection Biotin Binder kit (Invitrogen), or panning with purified anti-CD25 mAb. The enriched CD25^+^CD4^+^ cells were confirmed to be ∼95% Foxp3^+^. For flow cytometric analysis of tissue-infiltrating lymphocytes, mice were perfused transcardially with saline (0.9%) to remove intravascular peripheral blood lymphocytes. After various tissues were cut into small pieces, digested by collagenase (500 U/ml) and hyaluronidase (300 U/ml), and homogenized, the lymphocytes were purified by centrifugation with ficoll-paque (CEDARLANE Laboratories LtD., Burlington, NC).

### Confocal Microscopic Analysis

Frozen sections of lymph nodes were fixed with 3% paraformaldehyde in PBS, and pre-blocked with 1% BSA-2.5% FCS in PBS for 1 hour. Sections were stained with anti-mouse CD4 and Foxp3 (eBioscience, San Diego, CA) mAbs, anti-human CD4 (DAKO Japan, Tokyo, Japan), anti-human CCR7 (Abcam, Cambridge, MA or R&D Systems Inc., Minneapolis, MN) and biotin-conjugated anti-human Foxp3 (e-Bioscience) mAbs for one hour. After three washes in PBS, the sections were stained with Alexa Fluor 568 donkey anti-rat IgG (H+L) (Invitrogen), Alexa-Fluor 488 anti-goat IgG (H+L), Alexa-Fluor 568-conjugated streptavidin as the second antibodies for 30 minutes and washed with PBS. The nuclei were stained with 4′,6-diamidino-2-phenylindole (DAPI). The sections were visualized with a laser scanning confocal microscope (Carl Zeiss, Gottingen, Gremany). A 63×1.4 oil DIC objective lens was used. Quick Operation Version 3.2 (Carl Zweiss) for imaging acquisition and Adobe Photoshop CS2 (Adobe System, San Jose, CA) for image processing was used.

### Flow Cytometric Analysis

Surface markers were identified by mAbs with BD FACSCant flow cytometer (Beckman Coulter, Miami, FL). Rat mAbs of fluorescein isothiocyanate (FITC)-, phycoerythin (PE)-, or PE-Cy5-conjugated anti-CD4, and CD25, were used. Intracellular Foxp3 expression with an Intracellular Foxp3 Detection kit (eBioscience) was done according to the manufacturer's instructions. The data were analyzed with FlowJo FACS Analysis software (Tree Star).

### Migration Assay

To determine chemotaxis by S1P, CCL19, or CCL21 of Treg cells from WT and *CCR7* ^−/−^ mice, Cultrex 96 Well Cell Migration Assay Kit (Trevige, Inc., Gaithersburg, MD) was used in accordance with the manufacture's instructions. Prior to beginning assay, the cells were starved for 24 hours in the serum-free medium, and incubated in RPMI 1640 without FCS in the presence of S1P (0–100 nM), CCL19 (100 ng/ml), or CCL21 (100 ng/ml) with plate-coated anti-CD3 mAb (0.5 µg/ml) for 3–12 h.

### Human Samples

Immunostaining for CCR7 and Foxp3 was performed using lip biopsy samples from human SS patients and controls. All samples were obtained from the Tokushima University Hospital, Tokushima, Japan. All patients with SS were female, had documented as xerostomia and keratoconjunctivitis sicca, and fulfilled the criteria of the Ministry of Health, Labour and Welfare of Japan for the diagnosis of SS. All patients with SS had focus scores of greater than 2 by the Greenspan grading in their lip biopsy and all tested positive for autoantibodies against Ro. Analysis was performed under the certification of the ethics board of Tokushima University Hospital.

### Statistical Test

The Student *t* test was used for statistical analysis. Values of *p*<0.05 were considered as significant.

## Supporting Information

Figure S1 Change of CD25-CD4^+^ T cells of CCR7^−/−^ mice. The positive cells (%) of CD25-CD4^+^ T cells in spleen and LNs from WT and CCR7^−/−^ mice were analyzed by flow cytomery from 2 to 6 months of age. Data are means±s.d. of 6 to 8 mice per each group.
(0.05 MB PDF)

Figure S2 CCR7^+^Foxp3^+^ cells in the salivary gland tissues from SS patients. The expressions of CCR7 (red) and Foxp3 (green) of the infiltrating cells were analyzed by immunofluorescence staining. Nuclei were stained with DAPI. Representative photos are shown. The arrow heads show CCR7^+^ lymphocytes or dendritic cells.
(0.22 MB PDF)
