# Supplementary figures and images for: In Situ Patrolling of Regulatory T Cells Is Essential for Protecting Autoimmune Exocrinopathy
Source: PLoS One. 2010 Jan 5;5(1):e8588. doi: 10.1371/journal.pone.0008588 (PMC2798967; doi:10.1371/journal.pone.0008588)

Figure S1

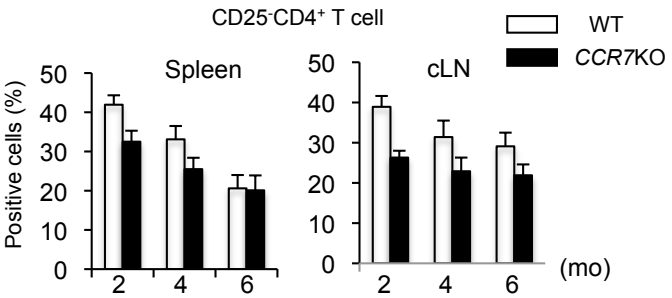

Supplement: Figure S1 — Change of CD25-CD4+ T cells of CCR7−/− mice. The positive cells (%) of CD25-CD4+ T cells in spleen and LNs from WT and CCR7−/− mice were analyzed by flow cytomery from 2 to 6 months of age. Data are means±s.d. of 6 to 8 mice per each group. (0.05 MB PDF) [file pone.0008588.s001.pdf]

Figure S2

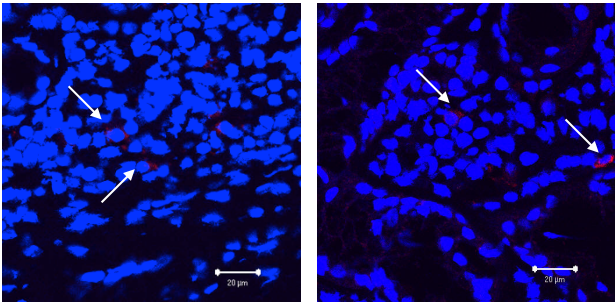

Supplement: Figure S2 — CCR7+Foxp3+ cells in the salivary gland tissues from SS patients. The expressions of CCR7 (red) and Foxp3 (green) of the infiltrating cells were analyzed by immunofluorescence staining. Nuclei were stained with DAPI. Representative photos are shown. The arrow heads show CCR7+ lymphocytes or dendritic cells. (0.22 MB PDF) [file pone.0008588.s002.pdf]
